# Beyond a viral succession timeline: a phase-transition framework and re-analysis highlight hidden instability in the proposed “phage clock”

**DOI:** 10.1128/aem.02307-25

**Published:** 2026-02-23

**Authors:** Nav La, Nathkapach K. Rattanapitoon, Chutarat Thanchonnang, Schawanya K. Rattanapitoon

**Affiliations:** 1 Faculty of Medicine, International University 231162, Phnom Penh, Cambodia; 2 FMC Medical Center, Nakhon Ratchasima, Thailand; 3 Mahidol University, Nakhon Sawan Campus 675179, Nakhon Sawan, Thailand; University of Nebraska-Lincoln, Lincoln, Nebraska, USA

**Keywords:** forensic microbiology, machine learning, metagenomics, viral-bacterial interactions, ecological phase transition, postmortem interval, virome succession

## LETTER

The recent article by Yu et al. presents important virome data across 35 days of cadaver decomposition and proposes that viral community profiles may provide a precise estimator of postmortem interval (PMI) (1). Their sequencing depth and temporal resolution are admirable and extend the microbial succession literature that has traditionally focused on bacteria and fungi. However, when these viral patterns are examined in the broader ecological context of decomposition processes described in landmark work on microbial community assembly, a different perspective emerges: the virome may not unfold as a stable clock but instead undergoes abrupt regime shifts that resemble ecological phase transitions rather than gradual chronological trajectories (2).

The patterns shown in the authors’ figures—together with the PERMANOVA results reported in Yu et al.’s supplemental Table S1—indicate marked restructuring of viral community composition around the mid-decomposition period. These discontinuities align with previously described threshold-dependent shifts during carcass breakdown and support the interpretation that virome dynamics may reflect ecological stage transitions rather than a smooth temporal gradient.

Additionally, the performance patterns shown in Yu et al.’s supplemental Table S2 demonstrate stage-dependent variability in prediction error. Such patterns mirror well-described limitations in microbiome-based PMI estimation frameworks, where predictive accuracy often declines when models encounter ecological restructuring or nonlinear turnover (3). The sensitivity implied by these stage-specific performance differences reinforces a recurring message in the forensic microbiome field: reliable PMI estimation depends not only on taxonomic markers but on understanding the mechanistic transitions that drive community reorganization (4).

The virome’s responsiveness to lysis–lysogeny cycling, nutrient pulses, and bacterial host fluctuations likely contributes to these abrupt transitions. In the absence of complementary functional indicators—such as absolute viral load, auxiliary metabolic genes, or integrase signatures—it remains difficult to determine whether observed viral patterns primarily reflect chronological progression or transitions between ecological regimes. Clarifying these mechanistic drivers would strengthen the interpretation of the temporal patterns and improve the translational potential of virome-based PMI estimation.

Our intention is not to diminish the contributions of Yu et al. On the contrary, their data set represents a significant advance and demonstrates the promising role of viral community data in forensic applications. We propose that situating these temporal patterns within a phase-transition framework—supported by the authors’ PERMANOVA results, variability in model performance across decomposition stages, and foundational decomposition ecology—may provide a more robust conceptual basis for future PMI models and help explain why high within-dataset accuracy may not consistently translate to broader forensic contexts.

## EDITOR'S NOTE

**Ed. Note:** The authors of the original article did not respond at the time of publication.
